# Dopamine regulates intrinsic excitability thereby gating successful induction of spike timing-dependent plasticity in CA1 of the hippocampus

**DOI:** 10.3389/fnins.2013.00025

**Published:** 2013-03-06

**Authors:** Elke Edelmann, Volkmar Lessmann

**Affiliations:** ^1^Institute of Physiology, Medical School, Otto-von-Guericke University MagdeburgMagdeburg, Germany; ^2^Center of Behavioral Brain SciencesMagdeburg, Germany

**Keywords:** dopamine, spike timing-dependent plasticity, action potential, β-adrenergic signaling, hippocampal slice

## Abstract

Long-term potentiation (LTP) and long-term depression (LTD) are generally assumed to be cellular correlates for learning and memory. Different types of LTP induction protocols differing in severity of stimulation can be distinguished in CA1 of the hippocampus. To better understand signaling mechanisms and involvement of neuromodulators such as dopamine (DA) in synaptic plasticity, less severe and more physiological low frequency induction protocols should be used. In the study which is reviewed here, critical determinants of spike timing-dependent plasticity (STDP) at hippocampal CA3-CA1 synapses were investigated. We found that DA via D1 receptor signaling, but not adrenergic signaling activated by the β-adrenergic agonist isoproterenol, is important for successful expression of STDP at CA3-CA1 synapses. The DA effect on STDP is paralleled by changes in spike firing properties, thereby changing intrinsic excitability of postsynaptic CA1 neurons, and gating STDP. Whereas β-adrenergic signaling also leads to a similar (but not identical) regulation of firing pattern, it does not enable STDP. In this focused review we will discuss the current literature on dopaminergic modulation of LTP in CA1, with a special focus on timing dependent (t-)LTP, and we will suggest possible reasons for the selective gating of STDP by DA [but not noradrenaline (NA)] in CA1.

## Introduction

Long-term potentiation (LTP) and long-term depression (LTD) are considered as neuronal substrates for learning and memory (see e.g., Whitlock et al., 2006). In comparison to high frequency stimulation or pairing protocols for **LTP induction**, and compared to prolonged low frequency stimulation induced LTD, **spike timing-dependent plasticity** (STDP) is dependent on only moderate numbers of repeated precisely timed single pre- and postsynaptic action potentials (APs) (for review see e.g., Duguid and Sjostrom, 2006; Caporale and Dan, 2008; Markram et al., 2011, 2012; Feldman, 2012). The availability of transmitter-type **neuromodulators** [such as DA, noradrenaline (NA), acetylcholine (ACh)] has been shown recently to crucially regulate STDP in different brain areas (Seol et al., 2007; Pawlak and Kerr, 2008; Zhang et al., 2009; Huang et al., 2012). Whereas these neuromodulators are known since a while to affect conventional LTP and LTD (for a recent review see e.g., Lisman et al., 2011), their cellular and molecular mechanisms of action in regulating **timing dependent (t-)LTP** and **t-LTD**, respectively, has just started to be investigated. In comparison to STDP in neocortex and striatum, the determinants of STDP in CA1 of hippocampal slices are less clear (see e.g., Buchanan and Mellor, 2010; Edelmann and Lessmann, 2011). In this focused review we will discuss the role of neuromodulators (especially DA) in the hippocampus for establishing STDP in the CA1 subfield of the hippocampus.

## Description of original discovery

To investigate STDP in the CA1 region, we applied whole cell patch clamp recordings from pyramidal neurons of acute hippocampal slices of rats and mice (for details, see Edelmann and Lessmann, 2011). Briefly, STDP was induced by extracellular stimulation of the Schaffer collaterals with a STDP paradigm consisting of one presynaptic and (mostly) one postsynaptic AP stimulation with a time delay of +5 to +10 ms. This stimulation was repeated 100 times every 2 s to induce timing-dependent (t-)LTP. We employed two different standard methods for slice preparation, using either sucrose-free artificial cerebrospinal liquid (ACSF), or sucrose containing ACSF (SUC). Using ELISA quantification our previous results revealed depletion of endogenous DA, selectively in SUC treated slices. Where indicated, DA levels were restored by bath application of exogenous DA (20 μM), or action of endogenous DA was blocked by application of a specific D1 receptor antagonist (10 μM SCH23390). Parallel to changes in synaptic plasticity we also monitored resting membrane potential and AP firing properties of the neurons. To analyze the specificity of DA effects on STDP, we applied in some experiments the β-adrenergic agonist isoproterenol (ISO, 10 μM).

**Figure 1:**
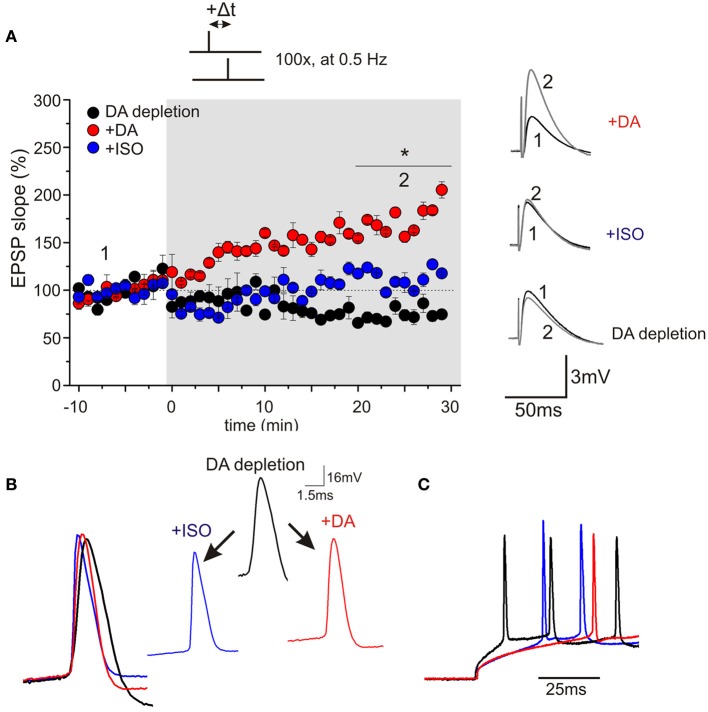
**Bath application of DA, but not ISO restores hippocampal spike-timing dependent plasticity in sucrose prepared slices. (A)** Synaptic responses were recorded in the current clamp mode in CA1 pyramidal neurons of juvenile rats (P15–P20). Pre-stimulus membrane potential prior to recording was −70 mV for all cells. Presynaptic stimulation of Schaffer collaterals was performed every 20 s (for graphs: data binned to 1 min means). After recording of baseline for 10 min, STDP was induced by a 1EPSP/1AP pairing (100×, every 2 s; at time point zero). Black symbols indicate neurons of the DA depleted group (DA depletion), red symbols indicate DA treated neurons (+20 μM DA) and blue symbols show ISO treatment (+10 μM ISO; ^*^p < 0.05). Inset on the left side shows original traces before and after STDP induction for the three different conditions. **(B)** AP rise times for all three conditions. Rise time is significantly faster after application of the neuromodulators. **(C)** Modulation of spike latency by neuromodulators: latency to first spike for DA depletion (black), rescue by DA (red) and unsuccessful rescue by ISO (blue).

**Figure 2:**
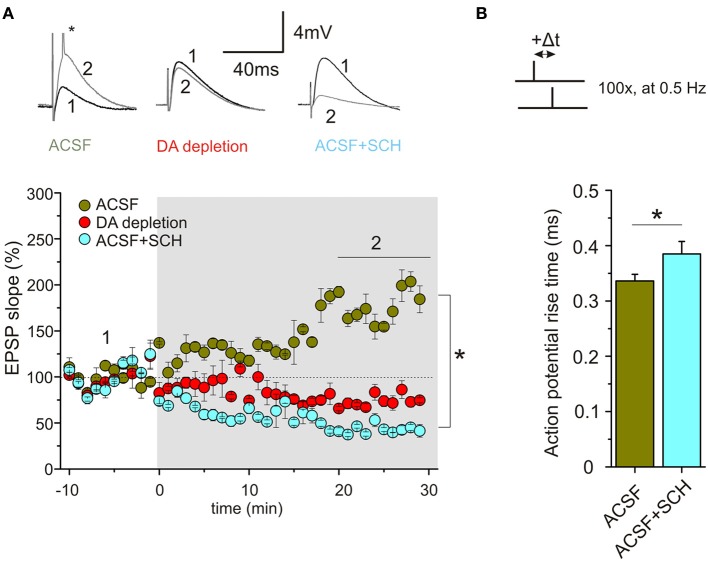
**Endogenous DA regulates STDP in CA1 of hippocampal slices via D1R signaling. (A)** Bath application of the specific D1R antagonist SCH23390 (10 μM) or DA depletion by preparation in SUC media inhibit t-LTP. For experimental conditions see Figure 1. Green symbols indicate average results for neurons of the ACSF group (ACSF), red symbols indicate results for DA depleted slices (DA depletion), and light blue symbols show results for neurons in ACSF prepared slices, treated with SCH23390 (+10 μM SCH). Inset above shows original traces before and after STDP induction for the three different conditions (small asterisk: action potential truncated). **(B)** AP rise time is significantly longer upon application of SCH23390 compared to ACSF (^*^p<0.05).

Using a STDP protocol with pairings of single pre- and postsynaptic spikes in CA1 pyramidal neurons of the hippocampus, our results revealed efficient induction and high amplitudes (on average 70% potentiation) of t-LTP when slices were prepared in ACSF solution, whereas preparation of slices in SUC containing solution strongly reduced the efficiency to induce STDP thereafter. ELISA measurements revealed reduced DA levels (to ~60% of ACSF slices) in SUC prepared slices. In parallel with reduced synaptic plasticity, spike rise times, firing latencies and accommodation properties during repetitive spiking, as well as rise times of APs, elicited by brief high amplitude current injections during STDP induction, were significantly changed in CA1 pyramidal neurons upon depletion of endogenous DA. Application of exogenous DA (20 μM) for 10–20 min restored all AP properties and also completely rescued t-LTP under these conditions, back to values as observed in ACSF control slices (Figure 1). Similar to DA, also application of ISO (10 μM), which has been shown previously to modulate backpropagating APs (Hoffman and Johnston, 1999), changed spike rise times (but not latency or accommodation). However, ISO was unable to rescue STDP (Figure 1). Interestingly, inhibition of D1 receptors in ACSF control slices inhibited t-LTP and also prolonged AP rise times (Figure 2). Overall, these results suggest that ambient levels of endogenous DA in hippocampal slices critically determine the efficiency of STDP induction in CA1, potentially by modulating properties of backpropagating APs during STDP induction (compare Edelmann and Lessmann, 2011).

## Discussion

### STDP in the hippocampus: under which circumstances can t-LTP be induced in CA1 pyramidal neurons?

Cellular mechanisms of LTP are best investigated for high frequency activation or theta burst stimulation of pyramidal neurons in CA1 of the hippocampus (Bliss and Collingridge, 1993; Malenka and Nicoll, 1999). The neuromodulator DA has been described to modulate the efficacy of high frequency stimulation induced LTP in CA1 of the hippocampus via D1/D5 receptor signaling (see e.g., Yang et al., 2002; Lisman and Grace, 2005; Swant and Wagner, 2006).

Because of the clear learning rules for the induction, and the limited numbers of stimulated presynaptic (usually <5) and postsynaptic (just 1) neurons, STDP is ideally suited to investigate the molecular mechanisms of synaptic plasticity in CA1 at the single cell level. STDP is dependent on precise timing of single presynaptic and postsynaptic APs at low frequency and repetition number, rather than on excessive repetitive synaptic activation (reviewed in e.g., Bi and Poo, 2001; Duguid and Sjostrom, 2006; Markram et al., 2011, 2012; Feldman, 2012). Positive timing (i.e., presynaptic activation precedes postsynaptic firing of an AP) leads to a t-LTP, while t-LTD is typically achieved if postsynaptic spikes repeatedly precede excitatory postsynaptic potentials (see e.g., Markram et al., 1997; Bi and Poo, 1998). Most STDP studies have been performed in the cortex, because cortical synaptic connectivity allows for local paired recordings of connected pyramidal neurons more effectively than the hippocampus with CA3 and CA1 pyramidal neurons being connected over long distances. Furthermore, the required pre- and postsynaptic firing patterns for STDP induction in CA1 are not well understood (compare e.g., Magee and Johnston, 1997; Harvey and Svoboda, 2007; Tanaka et al., 2008; Sugisaki et al., 2011). In dissociated cultures of hippocampal neurons, and in CA1 pyramidal cells of cultured organotypic slices (which—however—lack intact neuromodulatory inputs) STDP is well established (e.g., Debanne et al., 1994, 1996; Bi and Poo, 1998; Gerkin et al., 2007). In acute slices, STDP in CA1 is undisputed only in very young animals (Meredith et al., 2003, P9–P14 mice). For older, more mature preparations, the described learning rules in CA1 tend to be contradictory between different studies (for a recent review see Buchanan and Mellor, 2010). While Nishiyama et al. (2000) and Campanac and Debanne (2008) reported t-LTP after repetitive pairing of an EPSP with a single AP in CA1 of acute hippocampal slices from P26–P33 or P15–P20 rats, respectively, neither Pike et al. (1999, young adult rats), Wittenberg and Wang (2006, P14–P28 rats), Remy and Spruston (2007, 3–5 weeks old rats), nor Carlisle et al. (2008, 4–12 months old mice) could show t-LTP with single spike pairing conditions in CA1. It can be argued that species and age differences could account for these divergent results. However, even with a given species and age, and employing a similar STDP protocol, different learning rules have been described (compare Wittenberg and Wang, 2006; Campanac and Debanne, 2008). In the light of recent studies indicating a regulation of STDP either by DA in the striatum (Pawlak and Kerr, 2008; Pawlak et al., 2010), by ISO and ACh in the neocortex (Seol et al., 2007), or by DA in dissociated hippocampal cultures (Zhang et al., 2009), and given our own results of DA-dependent modulation of STDP in CA1 of hippocampal slices (Edelmann and Lessmann, 2011), we hypothesize that the availability of specific neuromodulators critically determine the efficacy of STDP induction in the hippocampus. Thus, the previous discordant results (see above and Table 1) for t-LTP in CA1 might be explained on the basis of differential availability of endogenous neuromodulators in hippocampal slices used in these studies.

**Table 1:** **Experimental conditions for hippocampal STDP at Schaffer collateral CA1 synapses in the hippocampus**.

**Study**	**Type of hippocampal preparation**	**Preparation condition**	**Species**	**Age**	**STDP protocol pre: post (current injection); repeat numbers with frequency or duration**	**Special conditions**	**t-LTP**
Debanne et al., 1994	Organotypic slice culture	Roller-tube culture	Rat	2–4 weeks (DIV)	1:>1 (240 ms, 0.5–2.0 nA); 50–100× ~0.3 Hz		Yes^*^
Debanne et al., 1996	Organotypic slice culture	Roller-tube culture	Rat	DIV>14	1:6–12 (240 ms, 0.5–2.5 nA); 50–100× at 0.3 Hz	Paired recordings	Yes^*^
Magee and Johnston, 1997	Acute slice	ACSF/sucrose?	Rat	5–10 weeks	5 × 5:5 × 1–3 (2 ms, 2 nA at 5 Hz); 2× at ~0.07 Hz	0.01 mM bic	Yes
Bi and Poo, 1998	Dissociated cell culture		Rat	DIV 8–14	pre:1 (1 ms, −70 to +30 mV); 60× at 1 Hz (post: suprathreshold EPSP response)	Paired recordings	Yes
Pike et al., 1999	Acute slice	ACSF	Rat	Young adult 120–200 g	1:1 (5 ms, 1 nA); 10× at 5 Hz	10 μM bic	No
					3:>1 (20 ms, 1 nA); 10× at 5 Hz		Yes
Nishiyama et al., 2000	Acute slice	ACSF	Rat	26–33 days	>1 (5 Hz):>1 (2 ms, 2 nA at 5 Hz); 16 s		Yes
			Mouse	20–23 days			
Meredith et al., 2003	Acute slice	ACSF/sucrose?	Rat	9–45 days	1:1 (5 ms, 0.1–0.85 nA); 30× at 0.2 Hz	9–15 days	Yes
			Mouse		1:1 (5 ms, 0.1–0.85 nA); 30× at 0.2 Hz	22–43 days	No
					1:1 (5 ms, 0.1–0.85 nA); 30× at 0.2 Hz +5 μM bic	30–45 days	Yes
					1:>1 (20 ms; 0.1–0.85 nA); 30× at 0.2 Hz	22–43 days	Yes
Wittenberg and Wang, 2006	Acute slice	ACSF	Rat	14–21 days	1:1 (3 ms, 1.2–2 nA); 70–200× at 0.1–5 Hz	100 μM PiTX	No
					1:2 (20–30 ms or 2^*^3 ms, 1.2–2 nA); 70–100× at 5 Hz		Yes
Remy and Spruston, 2007	Acute slice	ASCF	Rat	3–5 weeks	pre: 5 (100 Hz) inducing dendritic spikelets	4 μM SR95531	Yes^*^
					pre: 5 (100 Hz) without inducing dendritic spikelets	1 μM GCP52432	No
Harvey and Svoboda, 2007	Acute slice	Choline chloride	Mouse	14–18 days	1 uncaging stimulus:3 (2 ms, 1–3 nA, 50 Hz); 60× at 2 Hz		Yes
	Organotypic slice culture	Stoppini-culture	Rat	DIV 7–11			
Gerkin et al., 2007	Dissociated cell culture		Rat	DIV 10–15	1 (1–2 ms,100 mV):1 (1–2 nA, 2 ms); 60× at 1 Hz	Paired recordings	Yes
Campanac and Debanne, 2008	Acute slice	Sucrose-based ACSF (sodium-free)	Rat	15–20 days	1:1 (3–5 ms, 300–500 pA); 100× at 0.33 Hz	100 μM PiTX	Yes
Carlisle et al., 2008	Acute slice	ACSF	Mouse	6–12 months	1:1 (5–7 ms, 0.5–1 nA); 100× at 5 Hz	100 μM PiTX	No
				6–12 weeks	1:3–4 (50 ms, 0.5–1 nA); 100× at 5 Hz		Yes
Tanaka et al., 2008	Organotypic slice culture	Stoppini	Rat	DIV 8–12	MNI-glutamate uncaging (80 s at 1 Hz):1 (2 ms, 1–2 nA)		Yes
Hardie and Spruston, 2009	Acute slice	ACSF	Rat	4–8 weeks	1:1–3 (5 ms, 1.5–3 nA); 40× at 5 Hz	4 μM SR95531	Yes
					weak input: synaptically driven action potentials (1–3)	1 μM GCP52432	Yes
Zhang et al., 2009	Cell culture		Rat	DIV 9–15	1:1 (2 ms, 1–2 nA); 60× at 1 Hz	Paired recordings	Yes
Edelmann and Lessmann, 2011	Acute slice	ACSF	Rat	15–20 days	1:1 (2–3 ms, 1 nA); 100× at 0.5 Hz	100 μM PiTX	Yes
	Acute slice	Sucrose-based ACSF (sodium-free)	Rat	15–20 days	1:1 (2–3 ms, 1 nA); 100× at 0.5 Hz	100 μM PiTX	No
					1:2 (each AP: 2 ms, 1 nA); 50× at 0.5 Hz or 70× at 5 Hz		
					5:5 (each AP: 2 ms, 1 nA); 15–30× at 0.2 Hz		

The table compares preparation type, condition, species, age, and STDP protocol as well as the respective results in terms of induction of t-LTP. For interpretation of STDP protocol: 1:1 (2–3 ms, 1 nA); 100× at 0.5 Hz denotes one presynaptic stimulation (by extracellular stimulation, exceptions indicated) paired with one postsynaptic stimulation (induced by a 2–3 ms long 1 nA somatic current injection) repeated 100× at 0.05 Hz.

* Studies do not use the definition STDP or t-LTP; bic, bicuculline; GCP52432, GABA_B_ antagonist; PiTX, picrotoxtin; SR95531, GABA_A_ antagonist; >1, burst stimulation. (This table is an update of a previously published similar table in Edelmann and Lessmann, 2011).

### Neuromodulation of STDP

#### G-protein dependent modulation of t-LTP and t-LTD

Signaling via neuromodulators such as catecholamines (i.e., adrenaline, NA, DA) or ACh is known to be relevant for the efficacy of learning *in vivo* (e.g., Williams and Castner, 2006; Rossato et al., 2009), and for synaptic plasticity *in vitro* (e.g., Lisman and Grace, 2005; Scheiderer et al., 2008; Sara, 2009). Neuromodulator-dependent G-protein signaling regulates STDP in different brain areas.

In the visual cortex cooperation of different G-protein coupled receptors is necessary for functional bidirectional plasticity: exogenous ACh supports t-LTD via M1 receptors, whereas exogenously applied NA gates t-LTP via β-adrenergic receptors (Seol et al., 2007). The t-LTP promoting effect of NA relies on activation of β-receptors and subsequent Gs- and AC-signaling (Huang et al., 2012: but with pairing protocol instead of classical STDP; Salgado et al., 2012). Interestingly, while a high concentration of added NA enabled bidirectional STDP, low concentration of NA lead to a depression-only state (Salgado et al., 2012). Muscarinergic and α 1-adrenergic signaling via PLC- or Gq11 coupled receptors promote t-LTD and suppress t-LTP in the visual cortex (Seol et al., 2007; Huang et al., 2012). In the prefrontal cortex activation of nAChRs on GABAergic interneurons by exogenous nicotine was shown to block t-LTP via GABA_A_ receptor mediated attenuation of dendritic Ca^2+^ signals during t-LTP induction (Couey et al., 2007). Exogenous DA (via D2 receptors; D2R) enables t-LTP with intact GABAergic inhibition and prolongs effective spike timing windows for t-LTP by activation of D1/cAMP pathway (Xu and Yao, 2010). In the striatum, endogenous activation of D1/D5 receptors (D1/D5R) permits t-LTP and t-LTD, while inhibition of endogenous D2R signaling enables faster onset of potentiation in medium spiny projection neurons (MSNs) of the dorsolateral striatum (Pawlak and Kerr, 2008). In a related study, Shen et al. (2008) described distinct DA effects for a subpopulation of MSNs, which express D2R (but not D1R): for t-LTP, activation of the adenosine A2 receptor is necessary, while t-LTD can only be elicited after combined activation of the CB1 endocannabinoid receptor and D2 receptors. In the lateral amygdala, D2R activation by bath applied agonists permits t-LTP by decreasing feed forward inhibition (Bissiere et al., 2003), whereas in the ventral tegmental area (VTA) endogenous DA acts on D1 like receptors (most likely on D5R) to permit t-LTP in VTA DA cells (Argilli et al., 2008). Interestingly, the latter effect shares its signaling pathway with cocaine induced LTP in these neurons. Also in the hippocampus, neuromodulation of STDP is a common feature. Lin et al. (2003) reported an extended spike timing window for successful t-LTP induction after activation of β-adrenergic receptors, without affecting maximal t-LTP magnitude or t-LTP induction at short positive timings. In dissociated hippocampal cell cultures, Zhang et al. (2009) showed that exogenous DA via D1R activation prolonged the effective timing window for t-LTP, converted t-LTD to t-LTP, and reduced the threshold for induction of t-LTP. In dentate gyrus granule cells, activation of D1R by exogenous DA can reduce the threshold for t-LTP induction at medial perforant path-granule cell synapses in human and rat hippocampal slices (Hamilton et al., 2010; the authors used theta burst pairing instead of conventional spike timing protocols). At Schaffer collateral–CA1 synapses in acute rat hippocampal slices, D1R/D5R activation by endogenous DA is responsible for intact t-LTP at short positive pairings, and application of exogenous DA can rescue t-LTP after depletion of endogenous DA levels (Edelmann and Lessmann, 2011). However, activation of β-adrenergic receptors—although acting (like D1R/D5R) also via cAMP/PKA signaling—was not effective to restore t-LTP after DA depletion (Edelmann and Lessmann, 2011). For better comparison, the data discussed in this paragraph are summarized in Table 2.

**Table 2:** **G-protein dependent modulation of t-LTP and t-LTD**.

**Brain area**	**Study from**	**Neuron type**	**Involved neuromodulator (application)**	**Effect of neuromodulator**	**STDP protocol**	**Suggested mechanisms**
Visual cortex	Seol et al., 2007	Layer 2/3 pyramidal neurons	ACh via M1 Rs; NA via β-adrenergic Rs (agonist)	Bidirectional STDP only when neuromodulators act cooperatively	t-LTP: 1EPSP/4AP	AMPA receptor phosphorylation
					t-LTD: 4AP/1EPSP	
	Salgado et al., 2012	Layer 2/3 pyramidal neurons	NA via α- and β-adrenergic R (NA)	NA exerts dose dependent effects, gating of STDP	t-LTP: 1EPSP/1AP or 1EPSP/4AP	Unknown
Prefrontal cortex	Couey et al., 2007	Layer 5 pyramidal neurons	Nicotine via nAChRs (nicotine)	t-LTP is converted in t-LTD by nicotine	t-LTP: 1EPSP/1AP	Increase in GABAergic inhibition
	Xu and Yao, 2010	Layer 5 pyramidal neurons	DA via D1 and D2 receptors (DA and specific antagonist)	Permitting LTP by cooperative activation of D1 and D2 R, time window determined by D1 R	t-LTP: 1EPSP/1AP	Increase in GABAergic inhibition D1R unknown
Dorsal striatum	Pawlak and Kerr, 2008	Spiny projection neurons (SPNs)	DA via D1/D5 Rs (DA antagonist)	Normal t-LTP and t-LTD	t-LTP: 1EPSP/1AP	Unknown
					t-LTD: 1AP/1EPSP	
	Shen et al., 2008	SPN (subgroups)	DA via D1 and D2 R (DA antagonist)	Normal t-LTP and t-LTD	t-LTP: 3EPSP/3AP	Unknown
					t-LTD: 3AP/1EPSP	
Lateral amygdala	Bissiere et al., 2003	Projection neurons	DA via D2 Rs (DA and agonists)	Normal t-LTP	t-LTP: 3EPSP/3AP	Decreased feed forward inhibition
Ventral tegmental area	Argilli et al., 2008	Putative DA cells	DA via D1/D5R	Cocaine induced LTP is mediated by D5 R	t-LTP: 1EPSP/1AP	
			Cocaine injection blocks DAT transporter and thereby increase endogenous DA			
Hippocampus	Lin et al., 2003	CA1 pyramidal neurons	NA via β-adrenergic Rs (agonist)	Enhanced time windows for t-LTP, no effect on short timings	t-LTP: 1EPSP/1AP	Modulation of PKA and ERK signaling
	Zhang et al., 2009	Pyramidal neurons (cell cultures)	Dopamine via D1/D5 Rs (DA)	Enhanced time window for LTP, conversion LTD in LTD, facilitation of LTP	t-LTP: 1EPSP/1AP	Unknown
					t-LTD: 1AP/1EPSP	
	Hamilton et al., 2010	Granule cells (dentate gyrus)	DA via D1R (D1 agonist)	DA alter threshold for LTP	t-LTP: 4EPSP/4AP theta rhythms, theta-burst pairing	Via cAMP dependent increase in dendritic Ca^2+^
	Edelmann and Lessmann, 2011	CA1 pyramidal neurons	DA via D1/D5 Rs (DA and D1 antagonist) ISO via β-adrenergic R (agonist)	Normal LTP needs D1 signaling ISO not successful to restore LTP	t-LTP: 1EPSP/1AP	Modulation of action potential dynamics (ISO less effective?)

The table summarizes previously published studies focusing on dopamine dependent modulation of STDP in different brain regions (this table represents an update of a previously published table in Pawlak et al., 2010). ACh, acetylcholine, DA, dopamine; DxR, dopamine receptors, x, subtype; ISO, Isoproterenol; M1, muscarinic receptors, subtype 1; NA, noradrenaline; nACh, nicotinergic ACh receptor; R, receptor.

#### Modulation of ionic conductances by DA

Why is DA signaling essential for STDP? Neuromodulators such as DA have multiple effects on basal functions of neurons by different receptors coupled to different signaling cascades [**D1-like or D2-like receptor** (D1 or D2R), reviewed in Neve et al., 2004]. DA, acting on different subtypes of DR, can either increase neuronal excitability by release of Ca^2+^ from intracellular Ca^2+^ stores and via cAMP dependent mechanisms (e.g., Benardo and Prince, 1982; Lezcano and Bergson, 2002), or it can decrease excitability by reducing peak sodium currents, by means of cAMP dependent phosphorylation of α subunits of voltage gated Na^+^ channels (e.g., Cantrell et al., 1997). DA has also been described to either increase or decrease spike after hyperpolarization (e.g., Gribkoff and Ashe, 1984; Malenka and Nicoll, 1986; Berretta et al., 1990; Pedarzani and Storm, 1995), and to modulate further neuronal ion channels. Thus, DA increases the activity of Ca^2+^ activated K^+^ channel (Benardo and Prince, 1982; Pockett, 1985), activates dendritic voltage dependent K^+^ channels (Hoffman and Johnston, 1999; Johnston et al., 1999), increases h-channel activity (Rosenkranz and Johnston, 2006) and downregulates expression of t-type Ca^2+^ channels (Bender et al., 2010). Furthermore, DA can affect synaptic efficacy by changing frequency-dependent signal transmission via DA induced disinhibition (Ito and Schuman, 2008), or by enhancing net excitation by disinhibition of CA1 neurons via D3R (Hammad and Wagner, 2006). Since all of these DA-modulated voltage-dependent conductances contribute to shaping of APs, DA signaling likely modulates **backpropagation of action potentials** (bAP). This could account for the described DA-dependent alteration in the efficacy of STDP induction (compare section “G-protein dependent modulation of t-LTP and t-LTD”).

#### Mechanism of DA regulated STDP in the hippocampus

DA regulates conventional LTP in the hippocampus (reviewed in e.g., Lisman and Grace, 2005; Lisman et al., 2011). The main DAergic input to the hippocampus is delivered by VTA pathways (Gasbarri et al., 1994). DA via D1R activation can facilitate LTP induction by lowering the threshold to induce LTP (Li et al., 2003, 2011; Gao et al., 2006; Lemon and Manahan-Vaughan, 2006). Furthermore, endogenously released (by applying DA transporter antagonists) or exogenously applied DA, both, increase LTP in hippocampal slices (Swant and Wagner, 2006).

In our study (Edelmann and Lessmann, 2011), acute application of DA restored hippocampal STDP after DA depletion (~60% of control) during preparation of slices (see Figure 1). In parallel with the absence of functional STDP, we also observed changed firing behavior of postsynaptic pyramidal cells: latency to first AP was reduced, spike rise times were prolonged, and AP frequency was enhanced in DA depleted slices. Following application of DA, also AP parameters were completely restored. The DA application dependent changes in AP properties that we observed were most likely a consequence of complex modulation of various ion channels [i.e.; voltage gated Na^+^ currents, I_h_, K^+^ currents, T-type voltage gated Ca^2+^ channels (compare section “Modulation of ionic conductances DA”)]. Interestingly, application of ISO (β-adrenergic agonist) also restored most of the AP properties in DA depleted slices, but did not restore impaired STDP in our experiments. The noradrenergic (NA) innervation of the hippocampus *in vivo* is derived from the locus coeruleus (e.g., Scheiderer et al., 2008; Sara, 2009). Similar to DA transmission originating from the VTA, NA inputs in acute hippocampal slices are truncated and only axon shafts including presynaptic terminals remain in this preparation, which are likely to release DA and NA either spontaneously or in response to extracellular stimulation used to activate CA1 synapses. Our observation that a D1R antagonist (SCH23390) changes spike properties and inhibits t-LTP in CA1 are consistent with DA being released in the slices and thereby affecting firing properties and t-LTP. Whether effects of DA on spiking and backpropagation of APs can account completely for inhibited t-LTP after DA depletion, remains to be determined.

While activation of β-adrenergic receptors with ISO can restore STDP via adenylate cyclase (AC) signaling in the visual cortex (Seol et al., 2007), a similar rescue of STDP could not be detected in hippocampal CA1-STDP for short positive pairings (Edelmann and Lessmann, 2011). However, Lin et al. (2003) described β-adrenergic prolongation of spike timing windows for t-LTP induction in CA1. Possibly we were unable to see a rescue effect due to the short positive pairings used in our experiments. Alternatively, differential coupling of receptors to downstream signaling cascades and/or differences in subcellular localization of D1 vs. NA receptors could account for these seemingly divergent results between DA and NA signaling in t-LTP in CA1 (see e.g., Swanson-Park et al., 1999). It also seems possible that ISO concentrations which were sufficient for modulating firing of CA1 pyramidal neurons (Edelmann and Lessmann, 2011) were subthreshold for gating STDP. Thus, future analysis of dose response curves for DA and NA, respectively, should clarify whether DA and NA effects differ qualitatively or quantitatively in shaping t-LTP in CA1.

Differences in amplitude and time course of dendritic calcium waves might underlie the differential role of DA and NA, respectively, in gating STDP. Therefore, future calcium imaging in CA1 pyramidal neuron dendrites should be helpful to address this possible difference.

Importantly, only DA (but not ISO) changed AP latency and accommodation properties in our recordings from CA1 neurons, which could underlie the parallel gating effect of DA for STDP. Thus, future experiments investigating DA dependent modulation of K^+^ conductances which regulate these firing properties might be able to explain the differential modulation of STDP by DA and NA.

In conclusion, STDP studies should be evaluated critically for experimental conditions (such as preparation methods, age and species of animals, type of preparation, stimulation paradigms) which could result in changed availability of neuromodulators such as DA, NA, and ACh in the slices. From our own results and from several recent studies in striatum, neocortex and hippocampus we conclude that seemingly subtle differences in experimental conditions (e.g., in the composition of preparation and/or recording media) can dramatically influence the success of STDP. With respect to the action of a specific neuromodulator in STDP it seems highly relevant to investigate effects on basal and active electrophysiological properties, since changing AP properties and backpropagation of APs by specific neuromodulator actions might underlie changes in STDP.

### Conflict of interest statement

The authors declare that the research was conducted in the absence of any commercial or financial relationships that could be construed as a potential conflict of interest.

## Key Concept

LTP induction: Long-term potentiation (LTP) can be induced with various patterns of stimulation. Most used protocols are high frequency stimulations (HFS) or theta burst stimulations (TBS) to induce LTP or low frequency stimulation (LFS) to induce Long-term depression (LTD). More relevant to analyze signaling cascades during LTP is the paring protocol (combination of presynaptic tetanus and postsynaptic depolarization) or the low frequency stimulation protocol STDP.
Spike timing-dependent plasticity: Paradigm to induce synaptic plasticity. STDP is dependent on precise timed activation of pre- and postsynaptic cells. Physiological relevant amount of activation can be used to induce long-lasting plastic changes in synaptic transmission. This protocol works in patch clamp configuration and allows access to single postsynaptic cell.
Neuromodulators: Substances acting as neurotransmitters and modulate actions of multiple neurons and synapses. Most of those substances activate G-protein coupled receptors and alter synaptic transmission or ionic conductions. Examples for neuromodulators: dopamine (DA), noradrenaline (NA), acetylcholine (ACh), and serotonin (5-HT).
Timing dependent (t-)LTP: Timing-dependent (t-) LTP and LTD can be induced by STDP protocols. For t-LTP, presynaptic neurons have to be activated first, followed by the activation of postsynaptic neurons with ms scale delay. To evoke t-LTD, first postsynaptic neurons than presynaptic neurons are activated.
D1-like or D2-like receptor: Dopamine (DA) acts on two main types of DA receptors. D1-like receptors (consistent of D1 and D5 receptors) are coupled to adenylate cyclase (AC) by G-protein α_s_. D1-receptor activation lead to activation of AC and subsequent cAMP and PKA activation. D2-like receptors (D2, D3, D4) are coupled to inhibitory G-protein (Gα_i_), which reduce cAMP level.
Backpropagation of action potentials: Action potentials (APs) initiated at the axon hillock not only propagate into the axon, but also backpropagate into dendritic structures. Depending on the type of dendrite, this backpropagation is either regenerative (due to voltage gated channels along the dendrite) or decremental. In apical dendrites of CA1 pyramidal neurons, backpropagation is regulated by neuromodulators in a decremental fashion.
